# Use of Glycated Hemoglobin (A1c) as a Biomarker for Vascular Risk in Type 2 Diabetes: Its Relationship with Matrix Metalloproteinases-2, -9 and the Metabolism of Collagen IV and Elastin

**DOI:** 10.3390/medicina56050231

**Published:** 2020-05-11

**Authors:** Krasimir Kostov, Alexander Blazhev

**Affiliations:** 1 Department of Pathophysiology, Medical University-Pleven, 1 Kliment Ohridski Str., 5800 Pleven, Bulgaria; 2 Department of Biology, Medical University-Pleven, 1 Kliment Ohridski Str., 5800 Pleven, Bulgaria; yalishanda9@gmail.com

**Keywords:** type 2 diabetes, hemoglobin A1c, matrix metalloproteinases-2 and -9, anti-elastin antibodies, anti-collagen IV antibodies, diabetic retinopathy, diabetic nephropathy, macrovascular complications

## Abstract

*Background and objectives*: HbA1c measurements may be useful not only in optimizing glycemic control but also as a tool for managing overall vascular risk in patients with diabetes. In the present study, we investigate the clinical significance of HbA1c as a biomarker for hyperglycemia-induced vascular damages in type 2 diabetes (T2D) based on the levels of matrix metalloproteinases-2, -9 (MMP-2, MMP-9), anti-collagen IV (ACIV), and anti-elastin (AE) antibodies (Abs) IgM, IgG, and IgA, and CIV-derived peptides (CIV-DP) reflecting collagen and elastin turnover in the vascular wall. The aim is to show the relationship of hyperglycemia with changes in the levels of vascular markers and the dynamics of this relationship at different degrees of glycemic control reported by HbA1c levels. *Materials and Methods*: To monitor elastin and collagen IV metabolism, we measured serum levels of these immunological markers in 59 patients with T2D and 20 healthy control subjects with an ELISA. *Results*: MMP-2, MMP-9, and the AEAbs IgA levels were significantly higher in diabetic patients than in control subjects, whereas those of the AEAbs IgM, ACIVAbs IgM, and CIV-DP were significantly lower. MMP-9 levels were significantly lower at HbA1c values >7.5%. *Conclusions*: A set of three tested markers (MMP-2, MMP-9, and AEAbs IgA) showed that vascular damages from preceding long-term hyperglycemia begin to dominate at HbA1c values ≥7.5%, which is the likely cut-point to predict increased vascular risk.

## 1. Introduction

The prevalence of type 2 diabetes (T2D) is increasing worldwide, and it is expected to affect over 500 million adults worldwide by 2030 [1]. T2D is an important contributor to adverse cardiovascular complications, which are the leading causes of morbidity and mortality in Western countries [2].

Prevention of complications in T2D is closely linked to long-term control of hyperglycemia [3] since metabolic consequences extending beyond impaired glucose metabolism can affect almost every tissue and organ system of the body [4]. Despite the tendency in patients with good metabolic control to have a significantly reduced risk of developing complications, vascular disease can continue to develop and progress even under intensive treatment regimens due to the phenomenon known as “glycemic memory” [5]. Increased glucose levels can lead to metabolic derangements associated with vision loss, peripheral neuropathy, myocardial infarction, strokes, foot ulcers, and end-stage renal disease, which may cause permanent disability [6].

Despite the advancement of technologies to monitor blood glucose, for the vast majority of patients with diabetes, glycated hemoglobin (HbA1c) provides an excellent measure of glycemic control [7]. Nontraditional serum markers for short-term glucose control may enhance the ability to monitor hyperglycemia in people with diabetes. Fructosamine, glycated albumin, and 1,5-anhydroglucitol are of recent interest, especially in populations where the interpretation of HbA1c may be problematic such as in the setting of anemia, hemolysis, renal disease or pregnancy [8]. Most studies confirm a close linear relationship between HbA1c and mean blood glucose [9]. This suggests that HbA1c may be used not only as a diagnostic marker for the presence and severity of hyperglycemia during the preceding 4–12 weeks before the test but also over time as a “biomarker for a risk factor”, i.e., hyperglycemia as a risk factor for diabetic retinopathy (DR), diabetic nephropathy (DN), and other vascular complications [4]. A 1% increase in absolute concentrations of HbA1c is associated with about 10–20% increase in cardiovascular disease risk [10]. The American Diabetes Association (ADA) now recommends the use of HbA1c to diagnose T2D with a cut-off value of ≥6.5%. Individuals with HbA1c levels of 5.7–6.4% are considered to be prediabetic. The ADA also recommends in patients with T2D, values of HbA1c less than 7% to prevent long-term complications associated with the disease [11,12].

As in the general population, in patients with diabetes, the treatment and prevention of cardiovascular disease require the use of specific biomarkers to predict risk. Most of these biomarkers are focused on already known pathophysiological pathways and mechanisms affecting the cardiovascular system. In the diabetic population the advanced glycation end products (AGEs), endothelin-1 (ET-1), matrix metalloproteinases (MMPs), high-sensitivity C-reactive protein (hsCRP), N-terminal fragment of brain natriuretic peptide (NT-proBNP), high-sensitivity troponin T (hsTnT), lipids, and albuminuria can be useful in predicting of cardiovascular disease [13,14]. In this regard, the markers for glucose-induced vascular damage, such as AGEs and urinary microalbumin levels, may be particularly useful in predicting the risk in individuals with diabetes [13].

Important factors in the development of vascular complications in T2D are the increased glycation, degradation, and/or accumulation of elastin and collagen in the vascular wall [15]. MMPs, which hydrolyze the protein components of the vascular extracellular matrix, are actively involved in this process. The subgroup of MMPs known as gelatinases, in particular gelatinase A (MMP-2) and gelatinase B (MMP-9), can degrade collagen (COL), denatured COL (gelatin), elastin (EL), laminin, fibronectin, and other substrates [16]. Dysregulation of gelatinase activity is associated with vascular inflammation, remodeling, and fibrosis and may contribute to the pathophysiology of diabetic complications [17]. In a previous study of patients with hypertension and T2D, we showed that elevated serum levels of MMP-2 and MMP-9 may reflect early structural changes in the vascular extracellular matrix [14]. Unlike the other MMPs, MMP-2 and MMP-9 differ in that they contain three type II fibronectin repeats that have a high binding affinity for collagen. These repeats direct the catalytic pocket of the gelatinases close to the collagen, thereby enhancing the rate of their hydrolysis [18]. The enhanced proteolytic activity of MMP-2 and MMP-9 is accompanied by the release of COL, EL and their derivatives (e.g., EL-derived peptides (EDPs), COL type IV (CIV)-derived peptides (CIV-DP)) in blood circulation, which is followed by the production of specific anti-elastin (AE) and anti-collagen (AC) antibodies (Abs) from IgM, IgG, and IgA classes (AEAbs IgM, AEAbs IgG, AEAbs IgA, ACAbs IgM, and ACAbs IgG) against their epitopes. These autoantibodies can serve as valuable control biomarkers for the turnover of protein components in the vascular extracellular matrix (ECM). Elevated levels of anti-CIV (ACIV) Abs IgG (ACIVAbs IgG) in hypertensive patients with T2D may indicate increased degradation of CIV [19], which is the most abundant structural component in the basement membrane (BM) of the small vessels [20]. Similarly, elevated levels of AEAbs IgA may indicate increased degradation of the elastic fibers in the vessel wall as a sign of microvascular [21] and/or macrovascular [22] disease in T2D.

In the present study, we investigate the clinical significance of HbA1c as a predictive biomarker for hyperglycemia-induced vascular damages in T2D, based on the statistical relationships between HbA1c levels and corresponding levels of MMP-2, MMP-9, AEAbs (IgM, IgG, and IgA), ACIVAbs IgM, and the levels of CIV-DP, reflecting CIV and EL turnover in the vascular wall.

## 2. Materials and Methods

### 2.1. Study Population and Design

The study was approved by the University Research Ethics Committee and conducted in accordance with the Declaration of Helsinki (IRB approval no. 314-REC/Prot. 29). The study population consisted of 79 persons: 59 patients with T2D treated at the University Hospital Georgi Stranski, Pleven, and 20 healthy control subjects. Two groups were formed: Group I (*n* = 20): control group (Control); Group II (*n* = 59): patients with T2D. The clinical characteristics of the groups are shown in Table 1.

Selected control individuals were without diabetes mellitus, hypertension, or other vascular diseases, with a mean age of 61.5 ± 2.9 years. The mean age of patients with T2D was 60.7 ± 1.9 years. The patients were screened for microangiopathy using ophthalmoscopy and assessment of 24-h urine albumin excretion. Macroangiopathy was evaluated on the basis of clinical evidence for coronary artery disease, cerebrovascular disease, peripheral arterial disease, and/or history for acute arterial vascular events. Controls were screened for microangiopathy using ophthalmoscopy, and for macroangiopathy by physical examination, blood pressure measurement, electrocardiogram testing, measuring cholesterol levels, data on obesity and smoking, family history. The incidence of microangiopathy in the T2D group (*n* = 50) was 58%, and the incidence of macroangiopathy (*n* = 18) was 31%. Nine patients had both micro- and macrovascular diseases (Table 1).

According to the study design, our first aim was to compare the levels of MMP-2, MMP-9, AEAbs (IgM, IgG, and IgA), ACIVAbs IgM, and CIV-DP between patients and healthy controls. Our second aim was to compare within the patient group the levels of tested markers distributed below and above the different cut off values of HbA1c in the range between 6.0% and 8.0% (6.0%–6.5%–7.0%–7.5%–8.0%). All patients were divided into two subgroups according to these five cut-off values of HbA1c and we compared the levels of the markers between these subgroups (≤6.0% vs. >6.0%; ≤6.5% vs. >6.5%; ≤7.0% vs. >7.0%; ≤7.5% vs. >7.5%; ≤8.0% vs. >8.0%; see Table 2). 

### 2.2. Immunological and Biochemical Assays

All laboratory determinations were performed after 12–14 h overnight fasting. To measured the levels of MMP-2, MMP-9, AEAbs, ACIVAbs, CIV-DP, and the other laboratory parameters, blood was drawn into serum tubes. Serum was obtained after centrifugation at 2500 rpm for 10 min. Until the immunological assay, the serums were stored at −70 °C.

#### 2.2.1. Determination of MMP-2

To measure MMP-2 concentrations, an ELISA kit from R&D Systems (Cat. No. DMP2F0) (Minneapolis, MN, USA) was used. According to the manufacturer’s instructions, 100 μL of assay diluent RD1-74 was added to each well-plate, then 50 μL tested sera, diluted 1:10 with calibrator diluent RD5-32 (20 μL serum + 180 μL calibrator diluent) or standards, was added at various concentrations to construct a calibration curve. After 2 h downtime at room temperature on a shaker, plates were washed three times with 400 μL wash buffer per well. After the last wash, 200 μL of the conjugate was added to each well and incubated for 2 h at room temperature on a shaker. The plate was washed again three times, and in each well, 200 μL substrate solution was added. This was incubated for 30 min at room temperature in the dark. The reaction was stopped by adding 50 μL of stop solution to each well. Within 30 min, the serum samples were assayed at 450 nm on an automatic micro-ELISA plate reader (Coulter Microplate Reader UV Max).

#### 2.2.2. Determination of MMP-9

To measure MMP-9 concentrations, an ELISA kit from R&D Systems (Cat. No. DMP900) (Minneapolis, MN, USA) was used. According to the manufacturer’s instructions, to each well-plate, 100 μL of assay diluent RD1-34 was added, then 100 μL tested sera, diluted 1:100 with calibrator diluent RD5-10 (10 μL serum + 990 μL calibrator diluent) or standards, was added at various concentrations to construct a calibration curve. After 2 h downtime at room temperature on a shaker, plates were washed three times with 400 μL wash buffer per well. After the last wash, 200 μL anti-MMP-9 antibody conjugated with peroxidase was added to each well and was incubated for 1 h at room temperature on a shaker. The plate was washed again three times, and in each well, 200 μL substrate solution was added. This was incubated for 30 min at room temperature in the dark. The reaction was stopped by adding 50 μL of stop solution to each well. Within 30 min, the serum samples were assayed at 450 nm on an automatic micro-ELISA plate reader (Coulter Microplate Reader UV Max).

#### 2.2.3. Determination of AEAbs (IgM, IgG, and IgA)

To measure AEAbs IgM, AEAbs IgG, and AEAbs IgA concentrations, a sandwich ELISA was used. The assay was performed as follows: a microtiter 96-well polystyrene plate was coated with human aortic α-elastin, prepared as described by Baydanoff et al. [23] (1 μg of elastin in 100 μL of 0.05 M carbonate buffer, pH 9.6). Then the remaining “active” centers of the polystyrene wells were blocked by the plate incubation for 24 h with 1% solution of bovine serum albumin (BSA) (Cat. No. A2153, Sigma-Aldrich, St. Louis, MO, USA) in phosphate-buffered saline (PBS), pH 7.4, containing 0.05% Tween 20. The next step was the addition of 100 μL of tested patient serum (diluted 1:10 with PBS) in each well of the microtiter plate, incubated for 1 h at 37 °C. After washing three times, incubation with anti-human immunoglobulin peroxidase conjugates to the heavy chain of IgM, IgG, and IgA, respectively (Sigma-Aldrich, St. Louis, MO, USA) followed. All immunoconjugates were diluted 1:10,000 with PBS containing 1% BSA and 0.05% Tween 20. Next, samples were incubated with substrate solution (ortho-phenylene diamine, 4 mg/mL in 10 mL 0.05 M citrate buffer, pH 5.0 with H_2_O_2_) for 1 h at room temperature in a dark chamber. The reaction was stopped by adding 50 μL of 4 M H_2_SO_4_ to each well, and the optical density was measured with a micro-ELISA plate reader (Coulter Microplate Reader UV Max) at a wavelength of 492 nm. 

#### 2.2.4. Determination of ACIVAbs IgM

To measure ACIVAbs IgM concentrations, a sandwich ELISA was used. The assay was performed as follows: a microtiter 96-well polystyrene plate was coated with 100 μL of 10 μg/mL of human CIV (Sigma-Aldrich, St. Louis, MO, USA) at room temperature for 3 h, followed by overnight incubation at 4 °C. The plate was washed with phosphate-buffered saline (PBS) containing 0.05% Tween 20 and 1% bovine serum albumin (BSA; Cat. No. A2153, Sigma-Aldrich, St. Louis, MO, USA). Then, a 100-μL serum sample (diluted 1:10) was placed in each well of a microtiter plate and incubated for 1 h at 37 °C. After washing three times, 100 μL of goat anti-human IgM Ab, Fc5µ, HRP conjugate (AP114P, Sigma-Aldrich, St. Louis, MO, USA) were added to each well for 1 h at 37 °C. All immunoconjugates were diluted 1:10,000 with PBS containing 1% BSA and 0.05% Tween 20. The plate was incubated for 1 h at 37 °C. Ortho-phenylenediamine (4 mg/mL in 0.05 M citrate buffer, pH 5.0 with H_2_O_2_) was used as a colorimetric substrate. The reaction was stopped by adding 50 μL of 4 M H_2_SO_4_ to each well, and the optical density was measured with a micro-ELISA plate reader (Coulter Microplate Reader UV Max) at a wavelength of 492 nm.

#### 2.2.5. Determination of CIV-DP

To measure CIV-DP concentrations, a sandwich ELISA was used. The assay was performed as follows: each well of the microtiter plate was sensitized with 100 μL of 10 μg/mL of mouse monoclonal antibody to collagen IV (COL-94) (Cat. No. ab6311, Abcam, Cambridge, UK) at room temperature for 3 h, followed by overnight incubation at 4 °C. The plate was washed with phosphate-buffered saline (PBS) containing 0.05% Tween 20 and 0.1% bovine serum albumin (BSA) (Cat. No. A2153, Sigma-Aldrich, St. Louis, MO, USA). Then, a 100-μL serum sample (diluted 1:5) was placed in each well of a microtiter plate and incubated for 1 h at 37 °C. After washing three times, 100 μL of rabbit anti-human CIV polyclonal antibody (Cat. No. ab6586, Abcam, Cambridge, UK; diluted 1:2000) was allowed to react in each well at 37 °C for 1 h. The wells were washed with PBS + Tween 20, and peroxidase-conjugated goat anti-rabbit IgG H&L (HRP) (Cat. No. ab205718, Abcam, Cambridge, UK), diluted 10,000 fold, was then added to each well. The plate was incubated for 1 h at 37 °C. Ortho-phenylenediamine (0.4 mg/mL) was added to citrate buffer, and 100 μL of this solution was added to each well and allowed to react for 30 min. The reaction was stopped by adding 50 μL 4M H_2_SO_4_ to each well and the optical density was measured with a micro-ELISA plate reader (Coulter Microplate Reader UV Max) at a wavelength of 492 nm. 

#### 2.2.6. Biochemical Assays

The analysis was performed using an automatic biochemistry analyzer. HbA1c and CRP were measured by a turbidimetric immunoassay. Enzymatic methods were used to measure total cholesterol (TC), low-density lipoprotein cholesterol (LDL-C), high-density lipoprotein cholesterol (HDL-C), and triglyceride (TG).

### 2.3. Blood Pressure Measurements

Blood pressure (BP) was measured using a standard cuff mercury sphygmomanometer on the left arm in a sitting position, after 5–10 min rest. Normal BP was defined as SBP between 120 and 129 mmHg and/or DBP between 80 and 84 mmHg. Hypertension was defined as SBP ≥ 140 mmHg and/or DBP ≥ 90 mmHg, or if the patients have been diagnosed or had taken antihypertensive drugs at any time during the preceding six months.

### 2.4. Clinical Tests and Procedures

Each patient was subjected to the routine nephrologic (renal ultrasound, creatinine, blood urea nitrogen, urinary albumin excretion), ophthalmic (visual acuity test, ophthalmoscopy, tonometry) and neurologic (muscle reflexes, electromyography) examinations. Body mass index (BMI) was calculated using the standard metric BMI formula (Kg/m^2^). BMI between 18.5 and 24.9 was considered normal, 25 to 29.9 was considered overweight, and equal to or higher than 30 was considered obese.

### 2.5. Statistical Analysis

All statistical analyses were performed using the SPSS 23.0 software (SPSS, Inc., Chicago, IL, USA). The data were expressed as mean ± standard error of the mean (SEM) or standard deviation (SD) (in the figures) and were calculated at a confidence level of 95%. The differences between the groups were assessed by Student’s unpaired *t*-test. Correlation analysis was performed with Pearson’s correlation test. Values of *p* ≤ 0.05 were considered statistically significant.

## 3. Results

### 3.1. Comparison of the Tested Markers between the T2D and Control Groups

**Figure 1:**
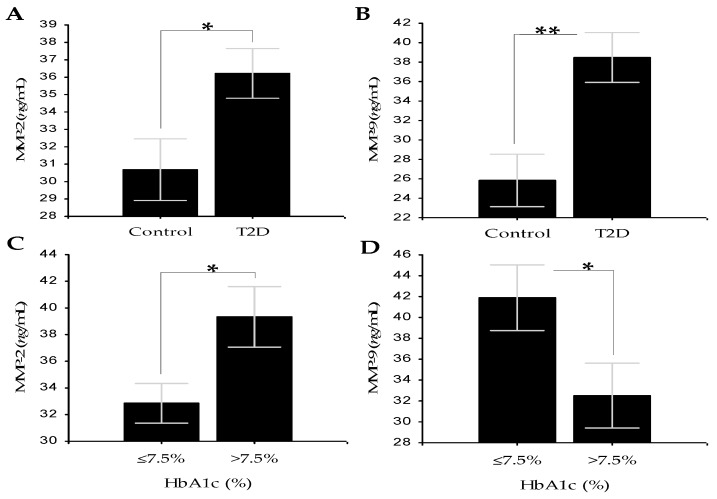
(**A**) Serum levels of MMP-2 in T2D group vs. control group. (**B**) Serum levels of MMP-9 in T2D group vs. control group. (**C**) Serum levels of MMP-2 in patients with HbA1c values ≤7.5% vs. patients with HbA1c values >7.5%. (**D**) Serum levels of MMP-9 in patients with HbA1c values ≤7.5% vs. patients with HbA1c values > 7.5%. Data are represented as mean ± SD. * *p* ≤ 0.05, ** *p* < 0.01.

**Figure 2:**
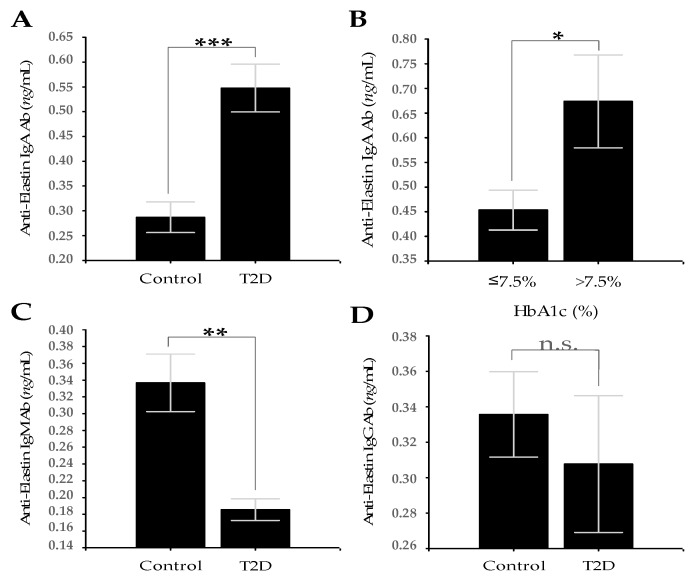
(**A**) Serum levels of AEAbs IgA in T2D group vs. control group (**B**) Serum levels of AEAbs IgA in patients with HbA1c values ≤7.5% vs. patients with HbA1c values >7.5%. (**C**) Serum levels of AEAbs IgM in the T2D group vs. control group. (**D**) Serum levels of AEAbs IgG in the T2D group vs. control group. Data are represented as mean ± SD. * *p* < 0.05, ** *p* < 0.01, and *** *p* < 0.001, n.s.—not significant.

Patients with T2D showed statistically significantly higher serum levels of MMP-2 (30.68 ± 1.87 vs. 36.22 ± 1.50; *p* = 0.026), MMP-9 (25.84 ± 2.83 vs. 38.48 ± 2.69; *p* = 0.002), and AEAbs IgA (0.29 ± 0.03 vs. 0.55 ± 0.05; *p* < 0.001) than healthy controls (Figure 1A,B and Figure 2A).

**Figure 3:**
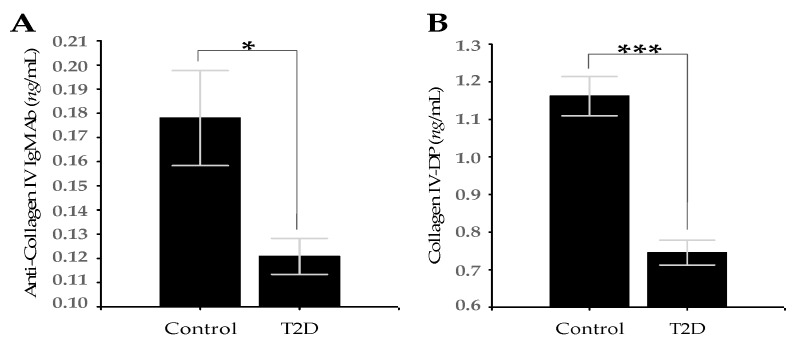
(**A**) Serum levels of ACIVAbs IgM in T2D group vs. control group (**B**) Serum levels of CIV-DP in T2D group vs. control group. Data are represented as mean ± SD. * *p* < 0.05, *** *p* < 0.001.

Serum levels of AEAbs IgM were significantly lower in T2D group than in controls (0.34 ± 0.03 vs. 0.18 ± 0.01; *p* = 0.001; Figure 2C). The levels of AEAbs IgG were also lower in the T2D group than in controls, but the difference was not statistically significant (0.33 ± 0.02 vs. 0.31 ± 0.04; *p* = 0.697; Figure 2D). The levels of ACIVAbs IgM (0.18 ± 0.02 vs. 0.12 ± 0.01; *p* = 0.016) and CIV-DP (1.16 ± 0.05 vs. 0.74 ± 0.03; *p* < 0.001) in patients with T2D were significantly lower than in controls (Figure 3A,B).

### 3.2. Comparison of the Tested Markers between T2D Subgroups at Cut-Off Values of HbA1c from 6.0 to 8.0%

Comparison of the tested markers levels at different HbAc cut-off values showed the most significant indication for vascular change at a cut-off HbA1c value of 7.5%. At this value, a set of three assessment markers for vascular risk (MMP-2, MMP-9, and AEAbs IgA) showed statistical significance (Table 2). In patients with poor glycemic control and increased vascular risk (*n* = 25), who have HbA1c values >7.5%, the levels of MMP-2 (32.85 ± 1.56 vs. 39.34 ± 2.39; *p* = 0.022) and AEAbs IgA (0.45 ± 0.04 vs. 0.67 ± 0.09; *p* = 0.049) were significantly increased compared to those with better control and HbA1c values ≤7.5% (*n* = 34; Figure 1C and Figure 2B). In the same subgroups of patients, MMP-9, unlike MMP-2, showed significantly decreased levels at HbA1c values >7.5% compared with HbA1c values ≤7.5% (41.89 ± 3.31 vs. 32.51 ± 3.26; *p* = 0.05; Figure 1D).

**Figure 4:**
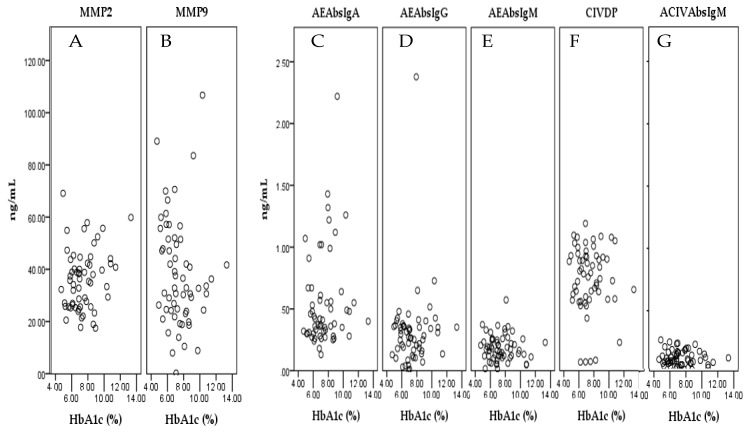
Scatterplots showing the relationship between the levels of (**A**) MMP-2, (**B**) MMP-9, (**C**) AEAbs IgA, (**D**) AEAbs IgG, (**E**) AEAbs IgM, (**F**) CIV-DP, (**G**) ACIVAbs IgM, and HbA1 as a continuous variable.

At the cut-off HbA1c value of 7.0%, statistical significance showed a set of two markers (MMP-9 and AEAbs IgA). MMP-9 showed significantly decreased levels at HbA1c values >7.0% compared with HbA1c values ≤7.0% (43.12 ± 3.45 vs. 32.19 ± 3.05; *p* = 0.023). The levels of AEAbs IgA were significantly increased at HbA1c values >7.0% compared with HbA1c values ≤7.0% (0.44 ± 0.04 vs. 0.66 ± 0.09; *p* = 0.031; Table 2). Only one marker (MMP-9) showed significantly decreased levels at cut off HbA1c values of 6.0% (50.79 ± 5.64 vs. 34.15 ± 2.40; *p* = 0.003) and 6.5% (46.42 ± 4.32 vs. 33.36 ± 2.65; *p* = 0.009). None of the markers showed statistical significance at a cut-off value of HbA1c of 8.0% (Table 2). The relationship between the levels of test markers and HbA1 as a continuous variable is shown in Figure 4.

### 3.3. Correlations of Investigated Immunological Markers

There were significant correlations of the examined markers for vascular risk in the T2D group, which are presented in Table 3.

## 4. Discussion

HbA1c is major tool for assessing glycemic control and has strong predictive value for diabetes complications [24]. Given the broad informativeness of the test, it is imperative to know how it can be optimally applied to the management and assessment of overall vascular risk (micro- and macrovascular) in patients with diabetes [25]. Systematic review and meta-analysis of multiple databases suggest that in people with diabetes, the target levels for HbA1c to minimize vascular complications should range from 6.0 to 8.0% [26]. The UK Prospective Diabetes Study (UKPDS) [27,28] and the Kumamoto Study [29] confirmed that intensive glycemic control significantly decreased rates of microvascular complications in patients with T2D [24]. The Diabetes Control and Complications Trial (DCCT) [30], a prospective randomized controlled trial of intensive (mean HbA1c about 7%) versus standard (mean HbA1c about 9%) glycemic control in patients with T1D, showed that better glycemic control is associated with 50–76% reductions in rates of development and progression of microvascular complications. Epidemiologic analyses of the DCCT and UKPDS also suggest that further lowering of A1C from 7 to 6% is associated with further reduction in the risk of microvascular complications, although the absolute risk reductions become much smaller [24]. The Analysis of the Action in Diabetes and Vascular disease: Preterax and Diamicron Modified Release Controlled Evaluation (ADVANCE) study has shown that microvascular event risk begins above an HbA1c of 6.5%. For macrovascular event risk, inflection of the curve was seen at around 7%, and the risk increased at higher HbA1c levels [31]. In a cohort of patients with T2D in the UK, Currie and colleagues report that the ideal HbA1c level is 7.5% and that the recommendations should target such a value. Their analyses demonstrate that an HbA1c of approximately 7.5% was associated with the lowest all-cause mortality and the lowest progression to large-vessel disease events. An increase or decrease from this mean HbA1c value was associated with a heightened risk of adverse outcomes [32]. The data in this study are consistent with our results, which give the highest indication of vascular change (set of three assessment markers—MMP-2, MMP-9, and AEAbs IgA) at a cut-off HbA1c value of 7.5% (Table 2). Therefore, vascular damage from preceding long-term hyperglycemia begins to dominate at an HbA1c value greater than 7.5%, which is the likely cut-off point to predict increased vascular risk.

Current consensus-based guidelines do not fix one exact cut-point of HbA1c, beyond which vascular risk increases. All recommend individualizing HbA1c targets on the basis of patient characteristics. The ADA recommends the reasonable target of HbA1c for many non-pregnant adults to be less than 7%. An HbA1c of <7.0% may be targeted in the majority of patients, acknowledging the individual needs [12]. When a patient has just been diagnosed and is free from significant cardiovascular disease, the aim should be a range from 6.0–6.5%. By contrast, in an elderly patient with long-standing and/or complicated disease, relaxing the target to 7.5–8.0% may be wiser, given that the vascular benefits in terms of life expectancy are less relevant [24]. The American Association of Clinical Endocrinologists and American College of Endocrinology (AACE/ACE) guidelines recommend a target of 6.5% (if it can be achieved safely). The National Institute for Health and Care Excellence (NICE) guideline specifies 6.5% or 7%, depending on the patient’s treatment regimen. The Clinical Systems Improvement (ICSI) guideline recommends a target range less than 7% to less than 8% based on patient factors. According to recommendations of the American College of Physicians (ACP), for most patients with T2D, targets levels of HbA1c should be between 7.0% and 8.0% [6]. Our HbA1c cut-point of 7.5%, evaluated to predict higher vascular risk, falls in the middle of this recommended range.

When we compared the levels of MMP-2 and MMP-9 between the control group and the T2D group, we observed higher levels in patients with T2D (Figure 1A,B). As to our findings that MMP-2 and MMP-9 levels were significantly higher in T2D patients, similar findings have been reported by other researchers [33,34]. However, an impression in our study makes the observed opposite levels of the two gelatinases at HbA1c values greater than 7.5% (Figure 1C,D). In patients with poor glycemic control and increased vascular risk, who have HbA1c values >7.5%, MMP-2 levels were significantly increased compared to those with better control and HbA1c values ≤7.5% (Figure 1C). In the same subgroups of patients, MMP-9, unlike MMP-2, showed significantly decreased levels at HbA1c values >7.5% compared with HbA1c values ≤7.5% (Figure 1D). Similar results have been reported by Derosa and colleagues in children and adolescents with T1D. They reported that MMP-2 levels were significantly higher in patients with microangiopathic complications compared with control subjects and patients without complications. MMP-9 levels were significantly lower in patients with microangiopathic complications compared with control subjects and patients without complications. Based on these results, the authors postulated that MMP-2 may be a good index of the severity and stability of microangiopathy, and MMP-9 is a marker of macroangiopathy in diabetes [35]. We also found positive correlations between MMP-2 and AEAbs IgG and between MMP-2 and ACIVAbs IgM in the T2D group (Table 3). A possible explanation for this result is that MMP-2 may be involved in the process of elastin and collagen IV destruction and the development of vascular complications.

MMP-2 and MMP-9 play an important role in the development of microvascular and macrovascular complications in T2D patients. The recent results advocate that due to diabetes, the overexpression of MMP-2 and MMP-9 in the retina inhibits cell proliferation and differentiation and accelerates apoptosis, a phenomenon that precedes the development of histopathology characteristic of DR [36]. During the first stage of DR, increased retinal MMP-2 and MMP-9 enhance the permeability of the blood–retinal barrier via proteolytic degradation of tight junction protein occludin and disruption of the overall tight junction complex [37]. In addition, MMP-2 and MMP-9 facilitate apoptosis of retinal capillary cells and pericytes, which disrupts the normal vascular structure and leads to the formation of microaneurysms and hemorrhages [38]. During the advanced stage, MMP-2 and MMP-9 dissolve the vascular basement membrane and create the conditions for the formation of new vessels [39]. MMP-2 and MMP-9 are also implicated in the pathogenesis of diabetic macular edema and fibrovascular proliferation with tractional retinal detachment, which are the most common causes of vision loss in patients with DR [40,41,42]. The impact and contribution of MMP-2 and MMP-9 to the onset and progression of DN may be most critical in the earlier phases of the disease process, at a time in which enhanced matrix turnover, release of pro-fibrotic growth factors, and altered cell motility may damage the glomerular apparatus and tubular architecture [43]. In these phases of DN, MMP-9 can predict microalbuminuria several years before its appearance and can be prognostic marker for the renal involvement. In the late period of diabetes, decreased activity of MMP-2 and MMP-9 is observed with increased activity of tissue inhibitor of metalloproteinases-1 (TIMP-1). These leads to excessive deposition of type IV collagen and fibronectin in the BM and a decrease in effective filtration surface area [44]. In the advanced stage of chronic kidney disease, the activity of MMP-2 and MMP-9 is decreased and, in this late period, the fibrosis is difficult to reverse [45]. On the other hand, MMP-2 and MMP-9 are involved in the process of atherogenesis and development of arterial lesions in T2D [46,47]. They are synthesized in atheromatous plaques and are present at elevated levels in rupture-prone regions of arterial blood vessels. MMP-2 and MMP-9 were both correlated with plaque instability and there was a correlation between increased MMP-9 expression and cap rupture [48]. Plasma levels and zymographic activities of MMP-2 and MMP-9 are increased in T2D patients with peripheral arterial disease in comparison with healthy control subjects, and MMP-9 may be a useful marker for development of macrovascular complications in T2D [34].

EL fibers are essential structural elements of the vascular wall, especially of the arteries. They are considered the most resilient element of vascular ECM. The EL half-life is in the order of 40 years. [49]. Elastases are endopeptidases that cleave EL, resulting in the formation of EDPs. Elastases include serine- and cysteine- proteinases and four MMPs—MMP-2, MMP-9, MMP-7 (matrilysin), and MMP-12 (macrophage elastase) [50]. T2D is associated with an increase in the expression and activity of MMP-2 and MMP-9, and an increase generation of EDPs [51,52]. EDPs have immunogenic properties and favor the formation of specific AEAbs from IgM, IgG and IgA classes [53]. The presence of AEAbs can lead to the formation of circulating immune complexes and complement activation and K-cell-mediated antibody dependent cytotoxicity, which may further contribute to the destruction of EL in the arterial wall. This process can be maintained by specific T- and B-lymphocytes at sites of arterial damage [21,54,55]. When we compared the serum levels of AEAbs IgM, AEAbs IgG, and AEAbs IgA in T2D patients with those of non-diabetic subjects, we observed significant increases in AEAbs IgA (Figure 2A), whereas the levels of AEAbs IgM and AEAbs IgG were decreased (Figure 2C,D). This feature of the humoral immune response, with the prevalence of higher serum levels of general or specific IgA Abs in diabetic patients, is a generalized phenomenon documented in a number of studies [56,57,58,59]. In one of these, the patients with T1D and T2D with micro- or macrovascular complications have had higher serum IgA concentrations than the corresponding groups of patients without complications. Furthermore, the patients with three kinds of microangiopathy had slightly higher IgA levels than patients with only one kind; those with nephropathy and hypertension had even higher levels. The macroangiopathy groups have shown the highest IgA levels among the T2D subgroups with complications, and the lowest among the T1D subgroups. These data suggest that monitoring IgA may provide early warning of the possible presence simultaneously of micro- or macrovascular complications in T2D [56]. Studies in T2D patients have also shown that poor glycemic control may be associated with an increase in serum IgA Abs [60]. Our results provide compelling evidence for this and show that the levels of AEAbs IgA are influenced by the degree of glycaemic control reflect by measurement of HbA1c (Figure 2B). In patients with poor glycemic control and increased vascular risk, who have HbA1c values >7.5%, AEAb IgA levels were significantly increased compared to those with better control and HbA1c values ≤7.5%. Similar to total IgA [56], an increase in AEAb IgA levels may indicate increased degradation of EL in the vascular wall and may be a specific marker for micro- or macrovascular damage in T2D [21,22]. These findings are also supported by the positive correlations that we found between AEAbs IgA and AEAbs IgM, AEAbs IgG, ACIVAbs IgM, and the CIV-DP. AEAbs also showed significant negative correlations with SBP, DBP, and BMI in the T2D group (Table 3).

CIV represents up to 50% of all BM proteins [61]. Unlike fibrillar COLs of type I, II, and III, CIV forms a network structure and it is found to be crucial for vascular BM assembly and stability [62]. MMP-2 and MMP-9 can cleave most of the major macromolecules of the ECM, including COL types IV, V, VII, and X. Processing of CIV gives rise to the release of fragments that are able to behave as epitopes since they can be bound by circulating Abs [63]. Autoantibodies (autoAbs) against CIV are present in various inflammatory and autoimmune diseases [64]. This is the case of recurrent Goodpasture’s disease secondary to an autoreactive IgA Ab [63,65]. Moreover, autoAbs against CIV have been detected in children with T1D and vascular complications [66,67], as well as in hypertensive T2D patients with microangiopathy [19]. When we compared the serum levels of ACIVAbs IgM and CIV-DP, they were significantly lower in the patient group than in the control subjects (Figure 3A,B). A possible explanation for this result is that the levels and activity of MMP-9 in chronic hyperglycemia (HbA1c values >7.5%) are decreased, which leads to excessive deposition of CIV in the vascular BM and to its thickening [68]. Vascular BM thickening is the most characteristic structural abnormality of small blood vessels in DR and DN [69]. In addition, the highest HbA1c values exhibited the highest BM thickness in both the retinal and glomerular capillaries, which was observed in diabetic rats [70]. We also found positive correlations between ACIVAbs IgM and AEAbs IgM, between ACIVAbs IgM and AEAbs IgA, and between the CIV-DP and AEAbs IgA in the T2D group (Table 3).

Strengths of our study, unlike retrospective epidemiological studies, are that it reflects the direct relationship of hyperglycemia with vascular changes through the levels of appropriately selected biomarkers. Also, a design has been used in which the division of biomarkers into groups according to the degree of glycemic control (HbA1c levels) can provide valuable information on the vascular status of patients. A limitation of the study is the relatively small number studied persons, which requires these results to be confirmed in larger studies.

## 5. Conclusions

Considering the broad informativeness of the HbA1 test, it can be successfully applied to the management and assessment of overall vascular risk in patients with diabetes. Our results give the highest indication of vascular change (set of three assessment markers, MMP-2, MMP-9, and AEAbs IgA) at a cut-off HbA1c value of 7.5%. This indicates that vascular damage from preceding long-term hyperglycemia begins to dominate at HbA1c values ≥7.5%, which is the likely cut-off point to predict increased vascular risk.

## Figures and Tables

**Table 1:** Clinical characteristics of the groups in the study population.

Variables	Healthy Control Subjects	Patients with T2D
	(*n* = 20)	(*n* = 59)
Men, *n* (%)	10 (50.0)	25 (42.0)
Women, *n* (%)	10 (50.0)	34 (58.0)
Age, years ^1^	61.5 ± 2.9	60.7 ± 1.9
Duration of T2D ^1^	N/A ^2^	10.1 ± 1.0
HbA1c (%) ^1^	N/A ^2^	7.5 ± 0.2
BMI, kg/m^2 1^	24.9 ± 0.5	28.4 ± 0.5 ***
TC, mmol/L ^1^	4.2 ± 0.2	5.2 ± 0.2 *
LDL-C, mmol/L ^1^	2.8 ± 0.2	3.0 ± 0.1
HDL-C, mmol/L ^1^	1.2 ± 0.04	1.0 ± 0.03 ***
TG, mmol/L ^1^	1.4 ± 0.1	2.7 ± 0.4
CRP, mg/L ^1^	1.1 ± 0.2	8.4 ± 1.02 ***
Hypertension, *n* (%)	0 (0)	43 (73.0)
SBP, mmHg ^1^	121.5 ± 1.9	149.2 ± 1.7 ***
DBP, mmHg ^1^	78.2 ± 1.7	83.0 ± 1.5
Microangiopathy, *n* (%)	N/A ^2^	50 (85.0)
Macroangiopathy, *n* (%)	N/A ^2^	18 (31.0)
Neuropathy, *n* (%)	N/A ^2^	8 (14.0)

* *p* < 0.05, *** *p* < 0.001; ^1^ Mean ± SEM; ^2^ N/A, not available; BMI: body mass index; TC: total cholesterol; LDL–C: low-density lipoprotein cholesterol; HDL–C: high-density lipoprotein cholesterol; TG: triglyceride; CRP: C-reactive protein; SBP: systolic blood pressure; DBP: diastolic blood pressure.

**Table 2:** Statistical significance between the levels of test markers in T2D subgroups at cut-off HbA1c values of 6.0%, 6.5%, 7.0%, 7.5%, and 8.0%.

HbA1c Subgroups	≤6.0% vs. >6.0%	≤6.5% vs. >6.5%	≤7.0% vs. >7.0%	≤7.5% vs. >7.5%	≤8.0% vs. >8.0%
MMP-2	NS	NS	NS	S *	NS
MMP-9	S **	S **	S *	S *	NS
AEAbs IgM	NS	NS	NS	NS	NS
AEAbs IgG	NS	NS	NS	NS	NS
AEAbs IgA	NS	NS	S *	S *	NS
ACIVAbs IgM	NS	NS	NS	NS	NS
CIV-DP	NS	NS	NS	NS	NS

* *p* < 0.05, ** *p* < 0.01, NS—not significant; S—significant; MMP-2: matrix metalloproteinase-2; MMP-9: matrix metalloproteinase-9; AEAbs: anti-elastin antibodies; ACIVAbs: anti-collagen IV antibodies; CIV-DP: CIV-derived peptides.

**Table 3:** Pearson’s correlation coefficients and statistical significance between the variables in the T2D group.

Correlations	Correlation Coefficient	Statistical Significance
	*r*	*p*
MMP-2 vs. AEAbs IgG	0.273 *	0.036
MMP-2 vs. ACIVAbs IgM	0.343 **	0.008
AEAbs IgA vs. AEAbs IgM	0.327 *	0.012
AEAbs IgA vs. AEAbs IgG	0.500 ***	<0.001
AEAbs IgA vs. ACIVAbs IgM	0.365 **	0.005
AEAbs IgM vs. ACIVAbs IgM	0.679 ***	<0.001
AEAbs IgG vs. AEAbs IgM	0.308 *	0.017
AEAbs IgA vs. Systolic BP	−0.292 *	0.026
AEAbs IgA vs. Diastolic BP	−0.419 **	0.001
AEAbs IgM vs. Systolic BP	−0.306 *	0.019
AEAbs IgM vs. Diastolic BP	−0.263 *	0.045
AEAbs IgG vs. Systolic BP	−0.277 *	0.034
AEAbs IgG vs. Diastolic BP	−0.320 *	0.013
AEAbs IgA vs. CIV-DP	0.362 **	0.005
AEAbs IgA vs. BMI	−0.273 *	0.038

* *p* < 0.05, ** *p* < 0.01, *** *p* < 0.001; MMP-2: matrix metalloproteinase-2; AEAbs: anti-elastin antibodies; ACIVAbs: anti-collagen IV antibodies; CIV-DP: CIV-derived peptides; BP: blood pressure; BMI: body mass index.
